# A composite fishing index to support the monitoring and sustainable management of world fisheries

**DOI:** 10.1038/s41598-023-37048-6

**Published:** 2023-06-29

**Authors:** Yimin Ye, Jason S. Link

**Affiliations:** 1 Fisheries and Aquaculture Division, Food and Agriculture Organization of the United Nations, Rome, Italy; 2 National Oceanic and Atmospheric Administration, National Marine Fisheries Service, 166 Water Street, Woods Hole, MA 02543 USA

**Keywords:** Ecology, Conservation biology

## Abstract

Overfishing has severe social, economic, and environmental ramifications. Eliminating global overfishing is one of the United Nations’ Sustainable Development Goals (SDGs). The SDGs require effective policy and progress monitoring. However, current indicators are issue-specific and cannot be utilized to measure fisheries efficacy holistically. This study develops a comprehensive index that takes into account the inputs, outputs, and ecological implications of fisheries. These components are then merged to form a single composite fishing index that evaluates both total fishing pressure on the ecosystem and historical patterns. The global fishing intensity grew by a factor of eleven between 1950 and 2017, and geographical differences emerged. The fishing intensity of developed countries peaked in 1997 and has since fallen due to management, but developing countries’ fishing intensity has increased continuously over the whole research period, with quasi-linear growth after 1980. Africa has experienced the most rapid expansion in fishing activity and now has the highest fishing intensity. This index takes a more comprehensive and objective look at fisheries. Its worldwide spatial–temporal comparison enables the identification of similar temporal trends across countries or regions, as well as areas of uneven development and hotspot sites for targeted policy action.

## Introduction

Fisheries play a vital role in global food security, nutrition, livelihoods, and economies, generating 214 million tonnes of fish and employing 58.5 million people^1^, but they also have a significant impact on habitats, biodiversity, and aquatic ecosystem function^2^. Fisheries sustainability is threatened by overfishing and habitat deterioration^3^. How can fisheries meet the growing need for nutritious seafood for a growing global population, rising consumption levels, and dietary shifts while minimizing impacts to ecosystems? The United Nations' Sustainable Development Goals (SDGs^4^) established a goal for fisheries conservation and restoration to rehabilitate all overfished stocks to maximum sustainable yield (MSY) levels.

Unfortunately, despite some positive developments in nations such as Australia and the United States, the world's fisheries are drifting more and further away from the SDG target^5^. Joint efforts at the national, regional, and global levels will be necessary to attain the SDG Targets, with each responsible institution developing concrete policy paths and executing measures tailored to its own circumstances. These policies and strategies must address the primary drivers of fisheries and their impacts on ecosystems in order to be effective, and relevant indicators must be developed to aid in assessing policy and program effectiveness, as well as tracking progress toward the set goal of fishery sustainability.

Indicators are commonly used to inform decision making^6^. They have evolved into a vital tool for monitoring and public communication by simplifying a complicated picture through the key elements of an issue. Simplified and aggregated indices can be used to (i) aid in policymaking and the design of effective actions, used as policy decision criteria, i.e. reference points, (ii) enable measurement of performance and calibrating progress toward set goals, (iii) facilitate systematic comparisons to identify areas of uneven development and policy attention, and (iv) provide useful tools for communicating the accomplishments and requirements of policy reforms and practical action plans^7^.

Indicators are not new to fisheries and they are frequently used for revealing problems, identifying trends, activating regulations, and contributing to the process of priority setting, policy development and progress tracking^8^. For example, most fisheries management uses the MSY-based B/BMSY and F/FMSY indicators^9^, and the European Scientific, Technical, and Economic Committee assesses the Common Fishery Policy's success by estimating fish stocks with F > FMSY or spawning stock biomass less than BMSY^9^. The MSY-based stock status is also widely used as a criterion for defining fishery sustainability in international instruments such as UNCLOS^10^, the Fish Stocks Agreement^11^, and the Sustainable Development Goals^4^.

Yet MSY-based reference points are frequently concentrated on a particular, single fish species, with the broader ecosystem context and implications being largely disregarded^12^. Stock-based management has a number of serious ecosystem drawbacks. To begin with, managing all species at MSY levels would result in severe ecosystem overfishing, as a multispecies MSY is smaller than the sum of individual species’ MSYs (about 75%)^13,14^. Second, achieving MSY for all species in an ecosystem at the same time is impossible^15,16^, because each species has its own gear selection and effort level that can result in MSY. Third, many tropical fisheries are multi-gear and multispecies that can interact—intentionally or incidentally—with hundreds of taxa representing a range of species groups (e.g. elasmobranch, reptiles, teleosts) with vastly differing life histories and susceptibility to capture by these gears^17^. Hence, accurately monitoring and assessing all impacted taxa can be difficult, if not impossible, and cost-prohibitive, thus establishing fishery management measures that avoid excessive catch restrictions while protecting especially vulnerable species is a significant challenge^18^. Finally, single-stock assessments fail to account for the temporal dynamics of individual stocks' carrying capacity, which are frequently assumed to be constant over time but are influenced by stock interactions and ecosystem dynamics, especially in the face of climate change and species displacement and replacement caused by a variety of factors^19^.

The technical issues and operational limitations of single stock-based evaluation and management have been recognized since the 1980s, and the ecosystem approach has been championed as a solution^20^. Yet ecosystem approaches have proved to be slow and difficult to adopt, largely because of general lack of suitable management frameworks that can accommodate ecosystem elements, in particular scientifically defensible reference points that can be used to monitor and manage fishery impacts. To further explore ecosystem approaches, this study considers fisheries as a production system and creates three indicators based on inputs (*I*^*a*^) and outputs (*I*^*b*^) from fisheries, as well as the effects of fishing on ecosystems (*I*^*c*^), to quantify fishing pressure within an ecosystem. Input (*I*^*a*^) is measured by fishing effort, output intensity (*I*^*b*^) is defined as the ratio of catch to primary productivity in an EEZ, and ecosystem impact indicator (*I*^*c*^) tracks changes in mean catch trophic levels. The three indicators are then aggregated to provide a single composite fishing index (*FI*) that assesses overall fishery ecosystem performance and tracks historical trends without the need for substantial ecological modeling. The fishing index takes into account these three major dimensions of fishing, giving a more complete picture of fisheries. When combined with the three-dimensional indicators, the fishing index can be used to track both overall and specific aspects of fisheries performance, thus improving policy effectiveness, management efficacy, and public communication. It can also be used to rank countries, allowing for reporting and tracking of their fishing performance and changes, and therefore boosting accountability for improvement. Finally, a linear transformation function of the fishing index can be used to calculate the SDG Indictor 14.4.1—the fraction of fish stocks fished at biologically sustainable levels—and evaluate its progress.

## Results

### Global fishing pressure

**Figure 1:**
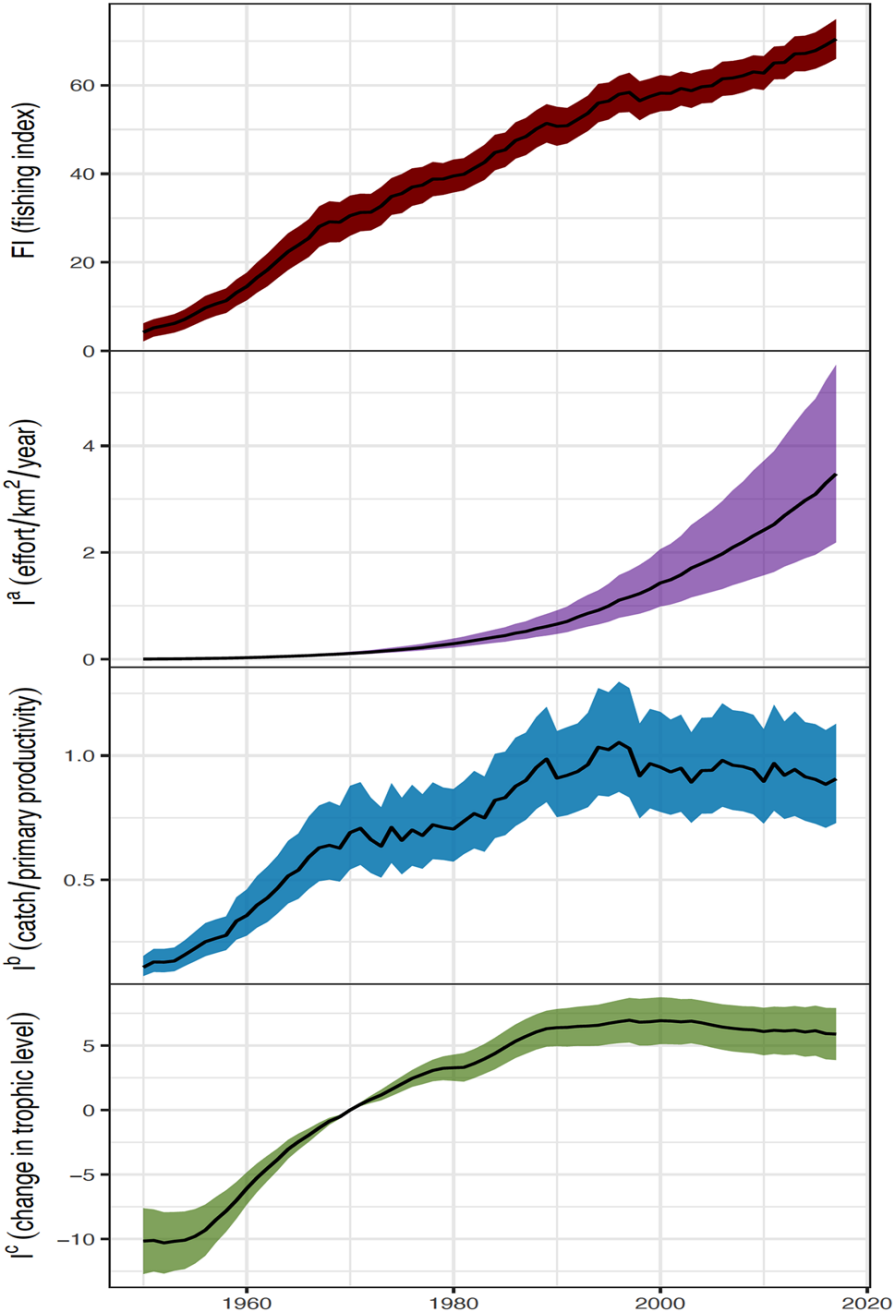
The global fishing index and three component indicators of fisheries from 1950 to 2017. The lines are geometric means weighted by EEZ’s average annual catches over the study period (Eq. 6) and the shadows are 95% confidence intervals. *FI* = the global index of fishing intensity; *I*^*a*^ = fishing effort per unit of area (kilowatt days/km^2^ per year); *I*^*b*^ = ratio (‰) of catch to primary productivity; and *I*^*c*^ = change in mean trophic level (percent) relative to 1970.

Between 1950 and 2017, the global fishing index (*FI*) increased by 11 times (Fig. 1). It increased linearly throughout the study period, with periodic declines from 1967 to 1997 and 1998 to 2017. Among the three component indicators, the input index *I*^*a*^ has increased exponentially from 0.001 to 3.474 kWd/km^2^ over the entire period (Fig. 1). The output index *I*^*b*^, on the other hand, was 0.11‰ in 1950, rose to 1.05‰ until 1996, and then fell back slightly to 0.91‰ in 2017. The ecosystem fishing impact (*I*^*c*^) peaked at 7% in 1997 when compared to 1970, then fell to 5.9% in 2017 (Fig. 1). It was at its lowest in 1950–55 (− 10% relative to 1970). In contrast to the input index, which continued to increase, both the output and impact indicators fell slightly after their peaks.

### Rankings of fishing intensity by ecosystem

**Figure 2:**
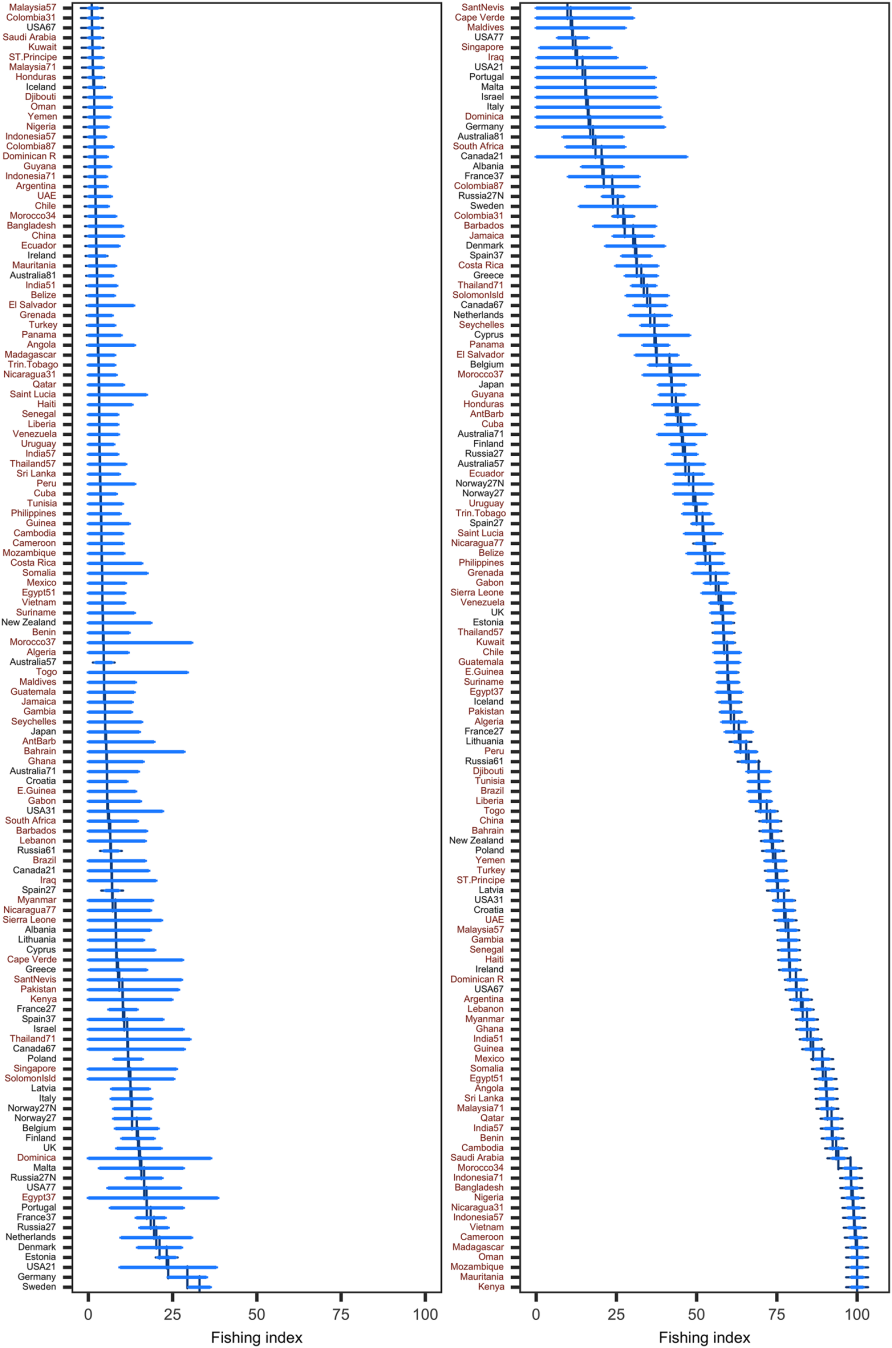
Comparison of fishing index estimations by EEZ-Areas in 1955 (left panel) and 2017 (right panel). Countries in black and brown are developed and developing nations, respectively. Vertical lines denote a EEZ’s mean of fishing index (*FI*), while bars indicate 95% confidence intervals.

Composite indices, which integrate multiple performance indicators into a single overall metric, are increasingly being used to rank systems based on their performance, allowing for the tracking of changes and the discovery of hot spots for targeted policy and management solutions. The 130 EEZ zones were ranked using the *FI* values in1955 and 2017. The EEZs with the highest fishing pressures in 1955 were predominantly from the developed world (18 out of the top 20 with *FI* > 15), which were mostly European countries and the United States (Fig. 2). Fisheries in developing countries were underdeveloped, 18 out of the last 20 EEZs (*FI* < 5) being in developing countries (Fig. 2). This situation has altered over time, and in 2017 it was totally reversed. All of the top 20 EEZs with the highest fishing intensity (*FI* > 75) are in developing economies, whereas only 8 out of the last 20 EEZs with the lowest fishing intensity (*FI* < 35; compared to 18 in 1955) are in the developing world (Fig. 2).

### Fishing in different geopolitical regions

**Figure 3:**
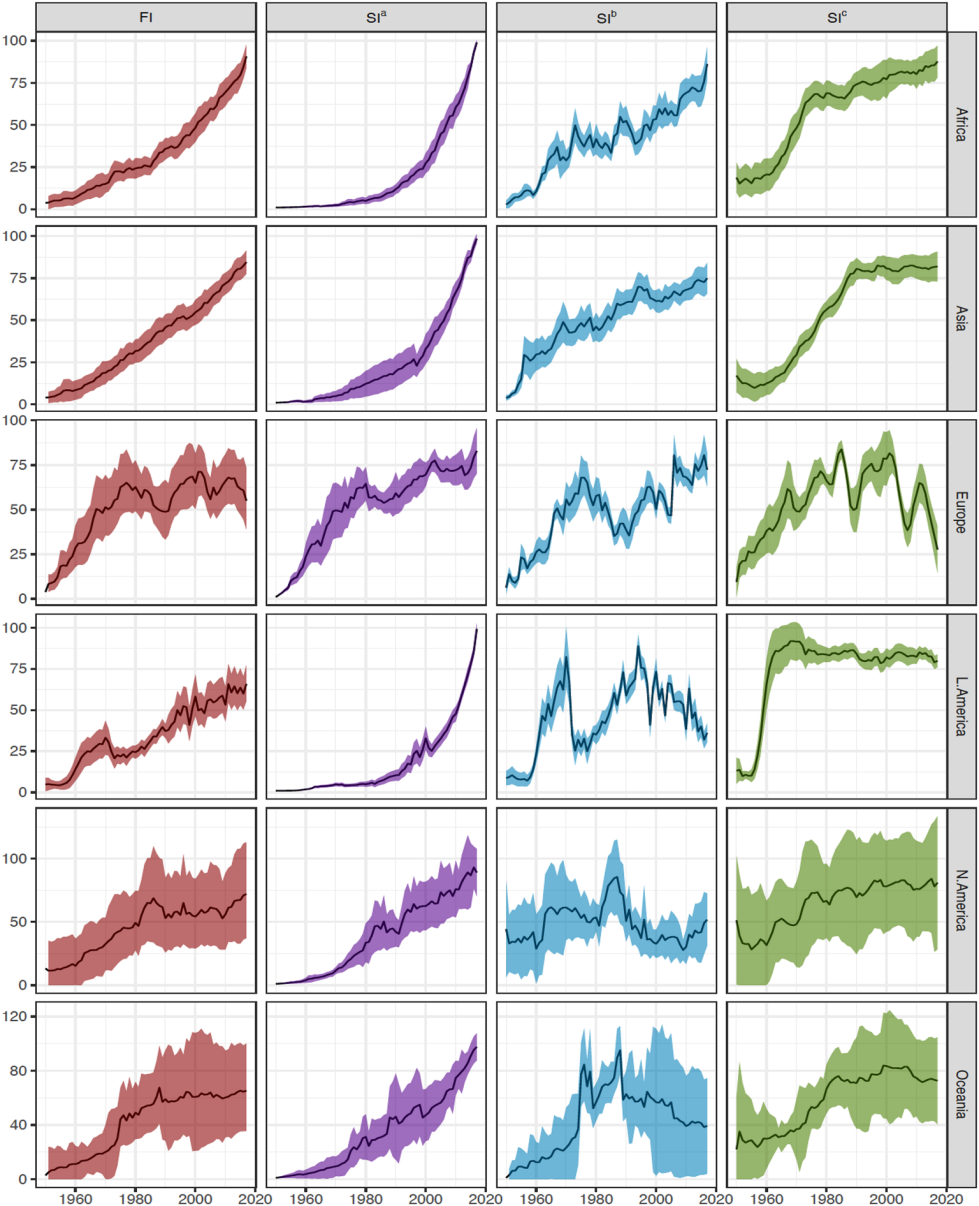
The estimated fishing index and three component indicators by continent. *FI* = the global index of fishing intensity; *SI*^*a*^ = standardized (see Eq. 6) input indicator; *SI*^*b*^ = standardized output indicator; and *SI*^*c*^ = standardized fishing impact indicator. Shaded areas indicate 95% confidence bands.

For statistical and political objectives, as well as the formulation and evaluation of policies, global concerns are analysed by continent. For each continent, we looked at the fishing pressure index and its component indicators. The six continents are divided into two groups by the fishing index. Europe, North America, and Oceania are examples of the first type, having grown in a hockey-stick pattern, initially expanding and then stabilizing at a specific level with oscillations (Fig. 3). The second category, which includes Africa, Asia, and Latin America, has continued to grow throughout the study period, with a notable dip in Latin America in the 1970s. In 2017, Africa had the highest *FI* score (90.8), followed by Asia (84.5), North America (72.0), Latin America (65.9), Oceania (65.3) and Europe (55.0) (Fig. 3). As a result, Africa not only has the highest average fishing pressure (1.7 times that of Europe), but it is also expanding at the fastest rate.

A thorough examination of the three component indicators reveals significant disparities in fisheries perspectives across continents (Fig. 3). While having the highest input intensity and fastest increase prior to 1980, Europe is the only region that has effectively controlled input pressure (*SI*^*a*^; which measures fishing effort per unit area) since 2003, though a bounce back seems clear over last two years. Over the course of the study, all other continents grew steadily, with Africa, Asia, and Latin America exhibiting exponential increase. In 2017, Europe had the lowest input intensity (82.9), followed by North America (89.1), and then the other continents (97.6–99.2) (Fig. 3).

*SI*^*b*^, which assesses the ratio of catch to primary productivity in an EEZ, exhibited three significant trends (Fig. 3). The first was a sustained rise in Africa and Asia, while the second was a decline in Latin America, North America, and Oceania following a peak in the 1990s or 1980s. Europe had peaked in the 1970s, dropped to a low in 1990, and then steadily rebounded with some turbulence. In 2017, Africa had the highest production intensity of 86.2 points, more than doubling that of Latin America (36.0), with Asia (74.8), Europe (72.4), North America (51.8), and Oceania (39) falling in between (Fig. 3).

Three patterns have emerged from the impact indicator (*SI*^*c*^), which monitors changes in mean catch trophic levels. Over time, both Africa and North America continued an upward trajectory, but this upward trend has slowed significantly since the 1970s (Fig. 3). While Oceania's decrease was small and uncertain, Europe and Oceania had both seen declines over the past two decades. After a period of significant increase, Asia and Latin America have remained largely constant since the 1980s and 1960s, respectively. In 2017, Africa had an *FI* 87.7, 60% higher than that of Europe (54.9), followed by Asia (81.9), North America (81.0), Latin America (79.9), and Oceania (72.6) (Fig. 3).

**Figure 4:**
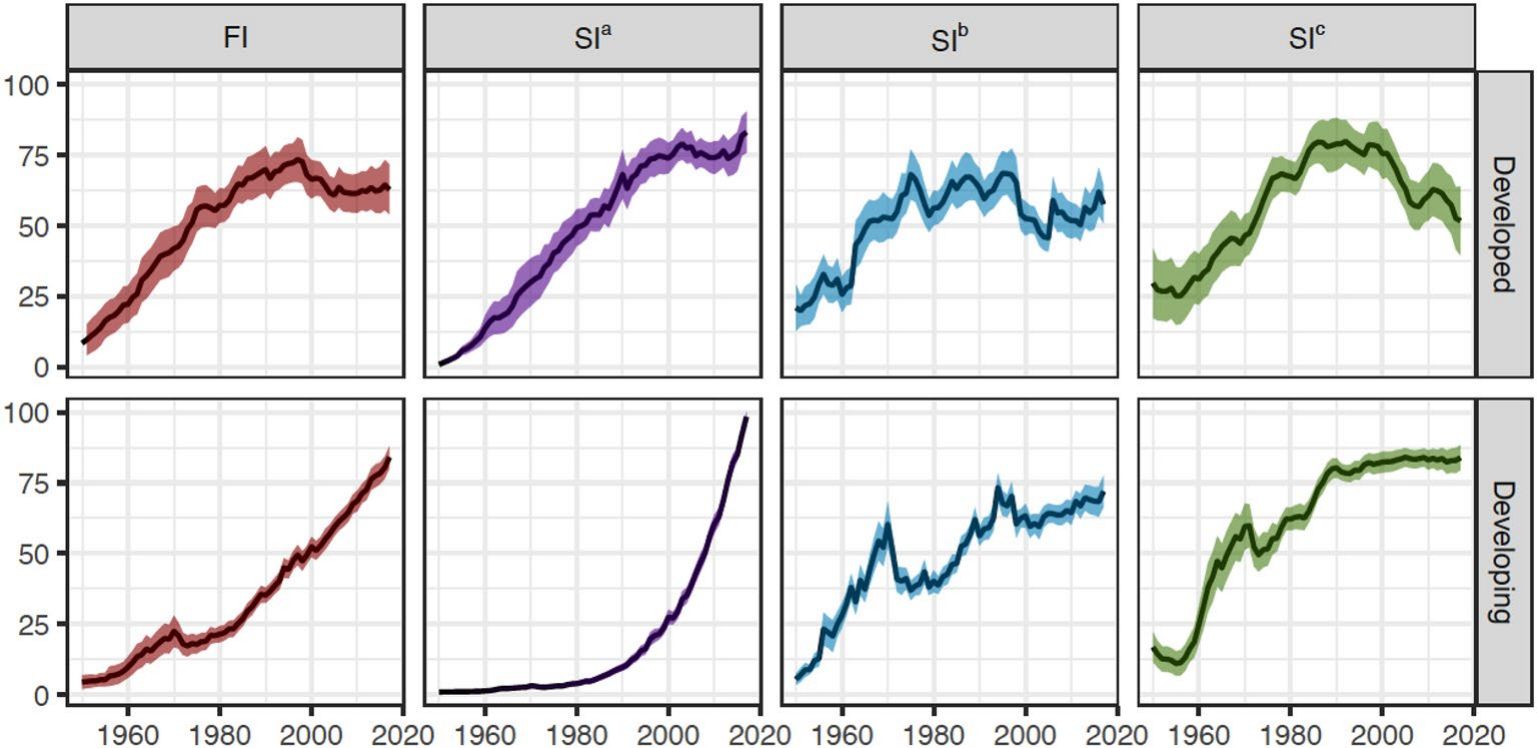
The estimated fishing index and three component indicators for the developed and developing countries. *FI* = the global index of fishing intensity; *SI*^*a*^ = standardized input indicator; *SI*^*b*^ = standardized output indicator; and *SI*^*c*^ = standardized impact indicator; Shaded areas indicate 95% confidence intervals.

The fishing index is grouped into two groups for a better understanding of their social and economic consequences: developed and developing countries. The difference between these two groups of countries was compared in Fig. 4. From 1950 to 1990, fishing pressure (*FI*) grew linearly in developed economies, reaching 73.5 in 1997 and then declined to 62.9 by 2017. In contrast, the *FI* in developing countries started at lower levels in the 1950s, increased slowly in the 1960s, dropped a small amount in the 1970s, but began to rise linearly afterward and shows no signs of slowing down. By 2017, the *FI* of developing economies has risen to 88.0, 22% higher than the developed world's (Fig. 4).

The input indicator (*SI*^*a*^) revealed a significant disparity between the two groups. *SI*^*a*^ in developed countries rose linearly until 2000, then stabilized, only with a resurgence in last two years (Fig. 4). Conversely *SI*^*a*^ in the developing world remained low until 1990 and has since exponentially expanded. The output indicators (*SI*^*b*^) for the two groups of EEZs, unlike the input indicator, have followed a similar developmental trend across time, growing and plateauing, but with a clear reduction after 2000 in the developed nations. While the developing world's impact indicator (*SI*^*c*^) has remained stable since 1990, the developed world’s has been falling downward since 2000 after an increase phase followed by a plateau (Fig. 4).

### Validity of the fishing index

A composite index’s ultimate evaluation is based on how well it measures what it purports to measure. However, because fisheries have multiple dimensions and the composite fishing index is not directly visible, verifying the validity of a composite index is particularly difficult. Most existing indices^21^ are meant to quantify only one aspect of a complicated process, and hence are not totally comparable to the composite fishing index. To see if the fishing index is viable as a measure of fishing pressure, we compared it to two existing indicators on fishery resources: the fish stocks indicator^[1]^ and the fraction of overfished ecosystems^22^. Because the fishing index’s operationalization procedure has no effect on these two indicators, this is an external validation, making it more objective and meaningful^23^.

The fishing stocks indicator (*FSI*) calculates the fraction of fish stocks with abundance equal to or greater than the maximum sustainable yield (MSY). To calculate this indicator, about 445 stocks around the world were assessed biannually using a tiered approach that includes formal model-based stock evaluation, data-limited approaches based on catch rates, trends, or surrogate measures of biomass, and expert judgment^24^. Fish stock is classified as biologically sustainable if its abundance is equal to or greater than the amount required to generate the maximum sustainable yield. When abundance falls below the MSY level, however, the stock is considered biologically unsustainable. The indicator was published biannually in the State of World Fisheries and Aquaculture^1^ and used to track progress toward the United Nations' SDG Target 14.4—successfully regulating harvesting and halting overfishing to restore fish populations to levels that can deliver MSY in the shortest period possible^4^.

**Figure 5:**
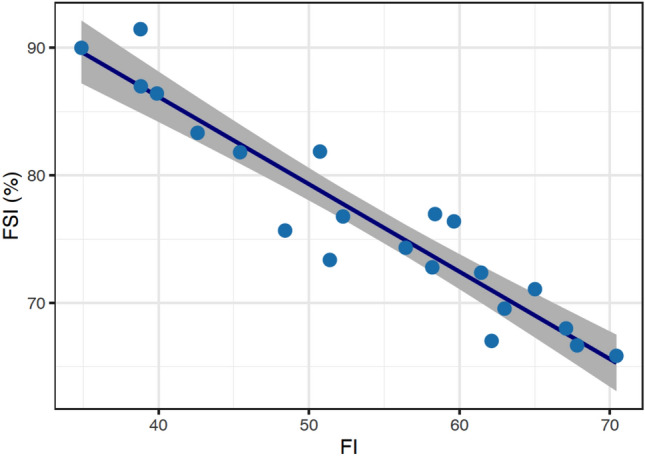
The relationship between the sustainability of fish stocks (*FSI*) and the fishing index (*FI*) from 1950 to 2017. The dots indicate data points, while the line is a linear model fit to the data with the shadows representing 95% confidence intervals.

The *FSI* measures the proportion of fish stocks fished at biologically sustainable levels^1^. In contrast, the fishing index (*FI*) measures the fishing intensity in a given area. A higher fishing index indicates a greater likelihood of overfishing. Thus, *FSI* and *FI* quantify two diametrically opposed phenomena: likelihood of vs risk to sustainability. If both indices are accurate measures of the critical features of fisheries, *FSI* should logically increase as *FI* decreases. A statistical model *FSI* = 117.2–0.8*FI* with *FSI* ≤ 100 was fit to show the relationship between the two. The model accounts for 89% of the variation in *FSI* observed (Figs. 5, S1, Table S1).

**Figure 6:**
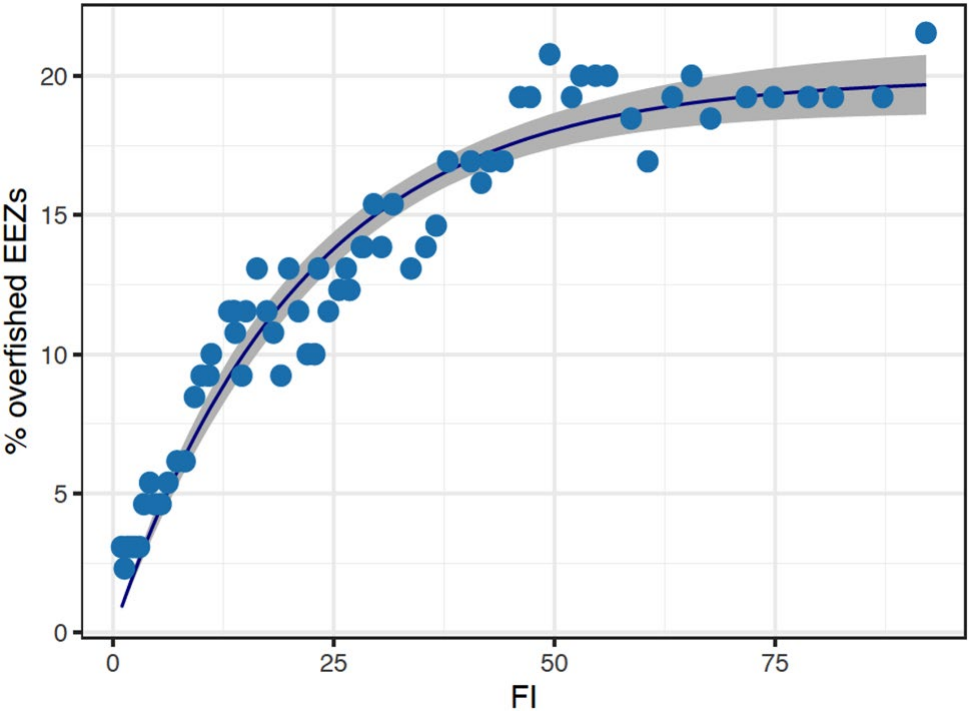
The relationship between the percentage of overfished EEZs and the fishing index (*FI*). The dots indicate data points, while the line is an exponential model fit to the data and the shadows represent 95% confidence intervals.

Ecosystem overfishing is widely acknowledged as the most significant issue to be addressed for the sustainable management of marine resources^25,26^. However, its precise definition remains elusive. The Ryther index, Fogarty index, and Friedland index are three measures proposed by Link and Watson^22^ to determine the occurrence of ecosystem overfishing. Due to data availability, we compared the *FI* with the Fogarty index (the ratio of total catches to total primary production in an ecosystem that exceeds 1‰ to evaluate the *FI*’s reliability in assessing whether an ecosystem has reached an unsustainable level of fishing. This ratio was calculated for all EEZs and the proportion of overfished EEZs (POE) was estimated for each year. The *FI* was found to be a good predictor for POE with *POE* = 19.9(1−e^− 0.05*FI*^). The correlation between these two indices was statistically significant (Table S1) and corresponds closely to the curvature of the data points (Figs. 6, S1).

Despite their inherent differences and the fact that they measure subtly different aspects of fishing, the *FI*’s strong relationship with percentages of overfished ecosystems (*POE*) and fish stocks sustainability (*FSI*) justifies its design and demonstrates its validity and reliability in measuring fishing pressure that is linked to the overfished status of fisheries, providing compelling conceptual and empirical evidence for the indicator's dependability and feasibility as a performance index of fisheries.

## Discussion

We present a unique methodology for creating a composite fishing index from three component indicators of fisheries, which can be used to measure performance and track progress toward policy and management goals. Our findings demonstrate that worldwide fishing intensity increased eleven times between 1950 and 2017 (Fig. 1). Divergent trends have emerged among geopolitical groups. The fishing intensity of developed nations peaked in 1997 and has since declined due to management, but the fishing intensity of developing countries has increased constantly during the whole research period, with quasi-linear increases after 1980 (Fig. 3). In the 1950s, the EEZs under the most severe fishing pressure were mostly in the developed world, but by 2017, the situation had completely flipped, with all of the 20 EEZs under the most severe fishing pressure being in developing economies (Fig. 2). Africa has had the greatest increase in fishing activity and today has the highest fishing intensity (Fig. 3). The historical and geopolitical trends and changes demonstrate varied levels of management achievement and suggest that developing countries have become priority areas for policy enhancement and action plans.

### Fishing pressure vs fishery performance

A fishery establishes a connection between ecosystems and society, with the primary objective of supplying food and providing social and economic services to humanity. Truly sustainable fisheries must be founded on three pillars: social, economic, and environmental sustainability. Each pillar should include indicators for monitoring the status and progress of fishery development^27^. This research focuses on fishing pressure on fishery ecosystems.

Fisheries are made up of various components. These components work together to determine the efficiency and environmental impact of fishing. Because of their simplicity, focus, and actionability, fisheries management tends to assess and control individual components of a fishery such as the number of vessels and total catch, and their performance is typically evaluated using component indicators linked with management objectives like F > F_MSY_ and B > B_MSY_^9^. Despite the fact that numerous indicators have been used to quantify fishing in relation to management goals, none are currently employed at the global level to consistently evaluate nations in order to report on their fishing pressure and track changes^28,29^.

This study developed a composite fishing index that integrates proxy information on three dimensions of fishery production: input, output, and ecological repercussions, in contrast to single-facet indices. It evaluated an ecosystem’s overall fishing effects and can help with the implementation of a whole-system approach to fisheries management. Its straightforward, standardized results can also aid communication with the public and decision makers, as well as facilitate comparisons between regions and countries in order to identify priority areas requiring policy attention and strategic action^30^. When used to rank countries, the fishing index can shed light on management efficacy, track progress over time, and promote accountability^31^.

The fishing index is a realistic attempt to measure and monitor the fishing state of national or even smaller-scaled fisheries, as it is easily estimable based on generally available data such as catch, fishing effort, and well-understood and accepted net primary production. Although the estimation and compilation of these key data required sophisticated computations and a large deal of labor, they have been made public and are constantly updated (see “Materials and methods”). With the proven effectiveness (Figs. 5 and 6), countries with limited data and capacity can use the availability of such data to develop the fishing index and its component indicators. This could help to address the world's fisheries' deteriorating sustainability, particularly in the majority of developing countries, which lack the essential data, technological competence, and resources for formal stock assessments^32,33^. It is worth noting that high variability in data quality and inter-country variation in indicator values may result in wider confidence intervals of regional estimates of indices, such as those shown in Fig. 3 for North America, which includes Bermuda, Canada, Greenland, Saint Pierre and Miquelon, and the United States.

As a dimensionless, quantitative metric, the fishing index enables comparisons of fishing pressure over time and across countries^34^. Its global spatial–temporal view permits the discovery of temporal trends across countries or regions (Fig. 2), as well as the identification of areas of uneven development and hotspot sites for focused policy intervention^35^. *FI* can serve as a variable of a function to estimate other indicators, as shown in Figs. 5 and 6, which require more expensive and technically demanding data collection. *FI* can also be combined with other indicators quantifying various aspects of fisheries, such as governance and compliance, to create an index evaluating the performance or sustainability of fisheries.

### Comparison with existing indicators

**Figure 7:**
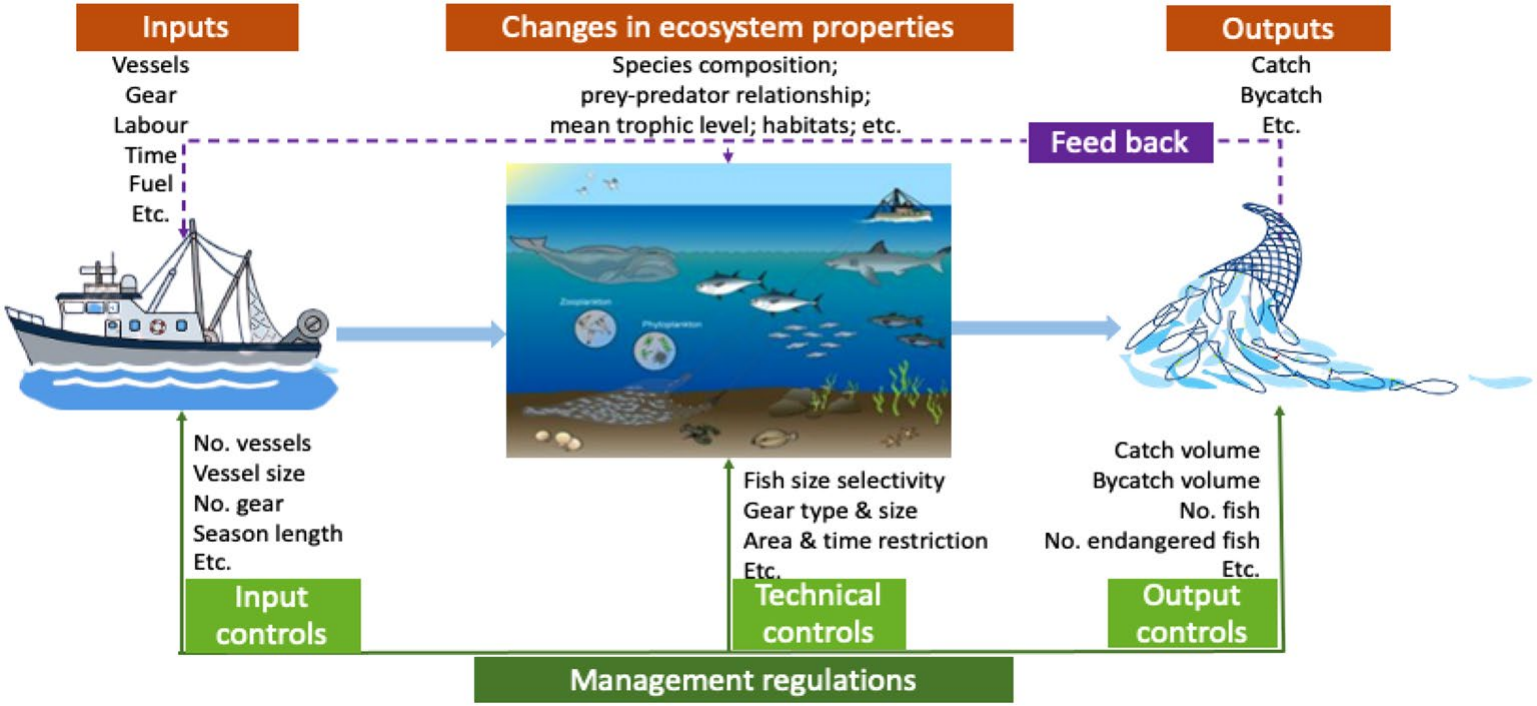
A diagram depicting a fishing system that includes inputs, outputs, and changes in the ecosystem’s features, as well as various control techniques extensively used in fishery management.

Despite the fact that there are numerous indicators that are pertinent to fisheries, none of them effectively reflect the multifaceted nature of sustainable fisheries and nor can they be used on a global basis. For instance, the size spectrum slope^36^, the primary production required by fisheries (PPRF)^37^, the Fishing in Balance Index (FiB)^26^, the percentage of primary production needed to sustain fisheries and the average trophic level of catch (percent PPR-TLc)^38^, the trophic spectrum^39^, the L-index^26^, and the likelihood that an ecosystem will be fished sustainably (Psust)^40^ are all indicators that only apply to the fishing impact or output components of the three dimensions examined in this study and are primarily concerned only with ecological perspectives on fisheries resources or the impact of fishing on ecosystems (Fig. 7). Second, these indicators require complicated data and are therefore difficult to use at the national level, especially in developing nations. Third, these indicators are inappropriate for assessing fisheries performance as a whole because they only measure specific aspects of fisheries. In contrast, the fishing index takes into account three components of fisheries: fishing activity (input), catch removals (output), and fishing-induced changes in ecosystems. These three factors are then combined to provide a comprehensive overall picture of fishing. Finally, the fishing index benefits from the use of openly accessible data and a simple computation, making it more approachable to nations with limited access to data and technical infrastructure.

Due to the global availability of common data and the notion of parsimony^41^, each of the fishing index’s three-dimensional indicators is composed of a single individual indicator. With the collection of additional data, each dimensional indicator in the current framework can be expanded to accommodate more individual indicators, allowing for improved measurement of multidimensional phenomena. Incorporating some of the indications described previously may further elucidate the indicator on ecological effects (i.e. impacts) of fishing.

### Contributing to the sustainable development goals

The United Nations’ SDGs seek to end overfishing by 2030, as shown by the percentage of fish stocks exploited at biological sustainable levels (Indicator 14.4.1). Despite the fact that a country is the basic unit of fishery management and regulations, this SDG indicator has only been analyzed at the global and regional levels^42^ due to technical and operational limitations. The validity of the composite index framework developed in this study is shown by the statistically significant relationships between the fishing index (*FI*) and the proportion of sustainable fish stocks (*FSI*). This relationship offers the possibility of estimating Indicator 14.4.1 from the *FI*, allowing for county-level assessments and assisting with SDG implementation.

Although the *FSI* is the most authoritative and widely accepted index among a number of existing indicators that attempt to monitor fishery sustainability^43^, it does not take into account any impacts other than those on a fish stock. As previously discussed, stock-focused indicators have technical limitations and confront operational challenges in multispecies fisheries, which are nearly the norm in practice. *FI*, on the other hand, assesses multiple dimensions of fishing pressure on ecosystems and is therefore thought to more accurately represent fishing impacts. The relationship between *FI* and the proportion of overfished ecosystems (Fig. 6) offers a method for estimating ecosystem overfishing based on fishing pressure. Although many indices have been devised to assess the effects of fishing on ecosystems, their specific data requirements have limited their global applicability. The use of publicly available data in the estimation of *FI* may facilitate its practical application and accelerate progress toward the Sustainable Development Goals by facilitating its estimation.

### Implications for policy

Global fishing intensity has steadily increased over time, which is consistent with the Food and Agriculture Organization's estimate of fish stock status decline^[1]^. The various trends across geographic regions indicate that Europe, North America, and Oceania have made headway in regulating fishing and its impacts on ecosystems, whereas Latin America, Asia, and Africa have been less effective (Fig. 3). Particularly in Africa, the exponential expansion of fishing creates a strong sense of urgency to manage its fisheries more sustainably. If fishing intensity is not lowered, fisheries production and economic and social efficiency would suffer. This will have a negative impact on the fisheries' long-term development. Thus, policy responses that are purposely developed for local situations and swift actions are essential in Latin America, Asia, and Africa. The shift in the EEZs with the highest fishing pressure may suggest that developing nations are a priority for policy reform and strategic planning.

Data collection, stock evaluation, regulations, fisheries management, and governance are all inextricably linked to social development. Regional disparities in fishery sustainability are primarily caused by economic and social development disparities. Most developing countries lack the infrastructure and technical expertise required to maintain an organized data collection system, conduct formal stock assessments, and develop and implement science-based management policies^32,44^. Developing countries must face these challenges on their own, but the international community must also help through global collaborations, initiatives, and other social and economic channels^32^, particularly those operating in developing countries^43^. We posit that the *FI* proposed here will help focus and simplify such efforts.

### Uncertainties, constraints, and future directions

Global indices, such as the *FI*, seek to quantify temporal advancement over time as well as regional heterogeneity. As a result, the indicators must be comparable both temporally and laterally across ecosystems. To that aim, a balance must be achieved between the functionality of an index and the availability of data to generate the index. Developing countries have only rudimentary, easily gathered fishing statistics, but they account for roughly 80% of global catches. To be inclusive, as recommended by the SDGs, this article proposes to construct indicators that use publicly available data rather than requiring large investments to collect newly purpose-designed data. Such an approach is bound to have limitations and ambiguities, which should be addressed here.

The output indicator is calculated by dividing the total catch by net primary production (Eq. 2). Primary productivity varies widely amongst ecosystems, typically by a factor of ten or more, therefore catch size alone is not a fair indicator of the impact of catch removals on an ecosystem, especially when comparing the most productive upwelling waters to the least productive open oceanic areas because ecological impacts of the same amount of catch removals differ greatly between the two types of ecosystems. The relationship between catch and NPP is frequently described as follows^45^, however with variations in detail in different studies

$$C=\alpha \times NPP\times {\left(\frac{1}{TE}\right)}^{{TL}_{eq}}$$

where *C* denotes catch, *NPP* denotes net primary production, and *TE* represents average transfer efficiency and *TLeq* is the equivalent trophic level of an ecosystem’s catch. The *C/NPP* ratio has been used as a proxy measure of the ecological impact of fish removals in numerous studies^26,37,46^ or to estimate fishery production potentials^47,48^ and other ecosystem related studies^22^. The ratio was employed as an index of fishing stress on the environment in this article.

However, the relationship between capture and primary productivity is confounded by uncertainty in the assessment of *NPP*, catches, fishing effort, and marine food web dynamics. Satellite remote sensing techniques are frequently used to generate *NPP* data^49^. Such information is available from a variety of public sources. It is worth mentioning that *NPP* estimations from different sources may differ slightly. To avoid bias, a study should use the same data sources for all countries. The *NPP* data utilized in this analysis is from the Sea Around Us project (https://www.seaaroundus.org/), and it changes with EEZ but is not time-dependent (Eq. 2).

It was estimated that approximately 20% of all catches were unreported globally^50^. To avoid underestimating the impact of fishing on the marine environment, complete extractions from the ecosystem, including illicit, unreported, and unregulated (IUU) catches, must be used^51^. The overall catch estimates used in this study are from Watson^52^ and are not just landings recorded publicly. The Sea Around Us project^53^ generated similar figures, which may differ somewhat. There is no preferred option here. It is critical to use a single dataset consistently throughout a study to ensure that results are comparable. If landings were employed instead of total catches, the resulting output index (*I*^*b*^) may be 20% lower on average. This underestimation will be greater in underdeveloped nations due to their higher IUU catches^50^.

The catch per unit effort (CPUE) statistic is widely used in fisheries and conservation biology. CPUE fluctuations are assumed to represent changes in the target species’ abundance^9^. It has proven to be useful in fisheries. However, CPUE is not used in this study because: (1) CPUE monitors primarily abundance, as opposed to the component indicators in this study that measure fishing pressure and thus does not fit in the design principles (see “Materials and methods” for details); (2) CPUE will diverge and move in a direction opposite to the fishing pressure indicators when fishing intensifies, making it difficult to aggregate into the *FI* index^30^; and (3) In general, CPUE data cannot provide the necessary information to analyze and manage communities or ecosystems^54^. To avoid misinterpretation, it is vital to distinguish between fishing pressure indicators and CPUE, which is not a simple derivative of the input (Eq. 1) and output (Eq. 2) indicators.

Fisheries managers may wish to establish whether the amount of *FI* is safe or causes concern for long-term sustainability. The action is fishing, and the consequence is sustainability. Both are made up of several components. *FI* summarizes the action side, but the outcome labeled as sustainability has yet to be summarized in a common fashion. The United Nations SDGs^4^, for example, utilize a proportion of biologically sustainable stocks as a gauge of sustainability, whilst others focus on avoiding ecosystem overfishing. After selecting a performance metric, the relevant *FI* reference value can be calculated. If the percentage of sustainable fish stocks is a concern, *FI* = 21.5 can be derived as the criterion using extrapolation, which generally includes significant uncertainty (Fig. 5). That is, all stocks are sustainable as long as *FI* is less than 21.5, but any further expansion will reduce the sustainability of fish stocks linearly. At *FI* = 70 in 2017, 65% of fish populations are biologically sustainable (Fig. 5). This example indicates that only after determining the effect of concern can a reference value of *FI* be calculated. There is presently no agreement on the metrics that can adequately describe fishery sustainability, and the objective of this study is not to establish a benchmark for *FI* in terms of sustainability, which will undoubtedly be a topic of future research.

The findings reported here should be considered with some caution due to some limitations. Indicator *I*^*c*^ tracks changes in the mean trophic level (MTL) of fisheries catches rather than the whole species in a marine ecosystem^55^ to monitor the effects of fishing on an ensemble of exploited species. Many indicators^26,37–40^ have been developed to monitor fishing impacts. Recently developed length-based indicators^56,57^, which demonstrate how stocks shrink on average as fishing pressure increases, can be beneficial in assessing fishing impact. Unfortunately, the bulk of these indicators are unreachable at the global scales and time series durations that we are attempting to achieve, and not all of them have universally relevant directionality with uniform interpretation. More work is required to systemically collect data that can support a more accurate assessment of fishing footprint, and the inclusion of such new data would increase the dependability and usage of these indicators, which can again be easily incorporated into *FI.*

The composite index *FI* integrates complicated, multi-dimensional fishing issues into a single aggregate number that can be used to help decision making, rank countries' performance, and highlight developmental patterns and hotspots for improvement^30^. Its simplicity can encourage public participation, raise public and stakeholder interest in the topics measured, and be used for policy advocacy. However, the integration process may result in the loss of essential information, and the building of a composite index involves subjective judgment in a number of processes such as aggregation, weights, and the filling of missing values. If a single composite index is not used correctly, it may be misinterpreted and lead to misleading policy messages to decision makers and the public, particularly if difficult-to-measure performance dimensions are ignored and the construction process is opaque and/or lacks sound statistical or conceptual principles^58^. In this study, the composite index is presented together with component indicators as a workaround.

## Materials and methods

A fishery is a sort of production system that uses fishing gear to catch fish in natural habitats. A production system's performance is frequently measured in terms of input intensity, output intensity, and system-level impacts^59^. In the fishing industry, inputs include vessels, gear, human labor, and so on, and outputs include catch, bycatch, and so on. Between the inputs and outputs is a series of fishing operations or activities that interact with and can modify fish stocks and the ecosystems that support them. As a result, it is critical to investigate the multidimensional character of the system and quantify three key components when measuring fishery performance: (i) system inputs, (ii) system outputs, and (iii) fishing-induced changes in the ecosystem’s properties.

The three aspects of a fishery production system are represented in Fig. 7, together with illustrative but not exhaustive major features and associated fishery regulating approaches. Although the three metrics, input, output, and fishing-induced changes, have historically been fundamental to traditional fishery management, fishery regulation is frequently centred on a single aspect, such as catch^60^, bycatch^61^, or species of conservation concern^62^ (output), vessel number, season length, effort, and accessible areas ^60^ (input), catch per unit effort^63^ (change in stock abundance), or habitat destruction (alteration to ecosystem)^64^. In contrast, this study examines the total performance of the three dimensions of fisheries.

The majority of existing fishery indicators are single-issue focused, such as the percentage of overfished stocks in a country or globally^1,65,66^, the net primary production required to sustain the current catch^37^, the marine trophic index^67^, or the fishing in balance index^68^. They simply reflect one part of fisheries' diverse nature, limiting their relevance in shapping policymaking and management methods^69^. This research generates several indicators of key fishing features and then integrates them in a systematic manner to produce a composite index for a holistic evaluation of a fishery production system^70^.

### Three indicators tracking inputs, outputs, and ecological effects

To design a comprehensive framework capable of quantitatively characterizing the performance of a fishing system, we must first determine the most influential elements affecting fisheries and their ecosystems. Catch, fishing effort^9^, target trophic level^71^, primary production^72^, water temperature^73^, and ecosystem type are all significant drivers of fisheries, according to Ye and Carroci^74^. These drivers are linked to the three dimensions of the fishery production system, except for the last two environmental components, which are uncontrollable (Fig. 7). The amount of input is defined by fishing effort (i.e., energy to capture fish over units of time), the scale of fishery output is measured by catch compared to primary production, and the trophic level indicates fishing-induced ecosystem changes. As a result, we use these key drivers to generate indicators of input intensity, output size, and ecosystem changes, which we then integrate into a single composite index assessing a fishery ecosystem's total fishing intensity.

In this study, an ecosystem as defined as the intersection of two geographic domains: flag states' exclusive economic zones and the FAO’s Major Fishing Areas for Statistical Purposes as by Ye and Carocci^74^. This is due to the fact that the state or its EEZ is the fundamental unit for collecting fishery data, developing policies, and enforcing management regulations, whereas FAO areas are defined by international fishery agencies based on a variety of factors such as statistical data collection, ecological differences, and jurisdictional purposes. The use of EEZ-Area intersections excludes catches caught in waters beyond EEZs that are mainly comprised of highly migratory species and meets the requirements for collecting data quickly, recognizing differences in the ecological characteristics of different drivers, and using analytical results to guide policy and management actions.

Vessels, gear, labor, and fishing time are all examples of anthropogenic fishing inputs (Fig. 7). In fisheries science, these inputs are usually standardized on their selectivity and efficiency to define input intensity. This is known as effective fishing effort, which is calculated by multiplying the quantity of engine power of fleets by the effective operational time^75,76^. The larger the fishing effort, the greater the fishing intensity, which subsequently influences catch, habitats and environmental repercussions. To make fishing intensity comparable across ecosystems, however, fishing efforts must be standardized by ecosystem area as well. Following Ye and Carocci^74^, this paper defines input intensity (*I*^*a*^) as the amount of fishing effort (*F*; kilowatt-days per year) expended per area of ecosystem (*A*; square kilometre) *i* during year *t*:

1 $${I}_{it}^{a}={F}_{it}/A_{i}$$

A fishery’s primary output is catch, which includes all species, including targeted, unintentionally caught or discarded (Fig. 7). The catch-to-biomass ratio is used in stock assessment to quantify fishing intensity at the output end^9^. However, because biomass accessible to fishing is difficult to estimate, net primary production is used instead, and the ratio (also known as the Fogarty ratio^22^) of total catches (*C*) to total net primary productivity (*P*) in an ecosystem^22^ is defined as an indicator that measures the output intensity (*I*^*b*^) of ecosystem *i* at year *t*:

2 $${I}_{it}^{b}={C}_{it}/P_{i}$$

Fishing alters the species composition, predator–prey relationships, fish habitats, and the ecosystem’s structure and function (Fig. 7). Changes in the environment and climate may have an effect on the species caught by fisheries. All of these changes are complicated and difficult to quantify, but they will affect both the mean trophic level of the catch and the environment. As a result, trophic changes have been frequently employed to evaluate the effects of fishing on aquatic ecosystems and the resulting changes in biodiversity^71,77^. We use changes in the mean trophic level (*L*) of fish species caught relative to mean trophic level in 1970 as a proxy indicator of fishing impacts (*I*^*c*^) on ecosystem *i* at year *t* in this paper

3 $${I}_{it}^{c}={1-L}_{it}/L_{i1970}$$

The mean trophic level is a complicated statistic that is influenced by a variety of factors, including changes in species composition caused by the environment, as well as interspecific predation and competition^49,78^. The trophic level of captures may temporarily increase if fishing fleets extend to deeper waters where species with high trophic levels are less fished and if technological advancements make high-trophic-level species more catchable^79^. In this study, the region-based mean trophic level^80^, which can correct for such bias, was used.

There are numerous potential indicators of ecosystem pressure in literature^26,37–40^. However, the majority of them necessitate extensive modeling, observations, or data, and the majority of those may be irrelevant to the vast majority of marine ecosystems. Despite the fact that it does not fully reflect changes in all trophic levels of a marine ecosystem in response to fishing influence, mean trophic level has been a popular proxy indicator used to examine fishing impacts on aquatic ecosystems and the resulting changes in biodiversity^26,81,82^. We believe that, despite the imperfect nature of the data, we should begin addressing real-world policy concerns with the best available information and continue to enhance it as technology and knowledge evolve.

The three indicators assess three different aspects of fishing. *I*^*a*^ measures fishing by the amount of effort expended on a unit area, *I*^*b*^ quantifies the amount of removed catches in relation to primary productivity, and *I*^*c*^ is concerned with changes in mean catch trophic levels—a proxy indicator of fishing-induced ecosystem changes. Any indicator with a higher value means a greater impact on the ecosystem. As a result, the three indicators collectively assess the overall intensity or pressure of fishing in each area.

The three indicators use different scales and units of measurement. Normalization is required when multiple indicators are utilized to create a composite indicator in order for them to be comparable and represented in the same units. This study employs the min–max normalization method^36,68,69^ as shown below:

4 $${SI}_{it}^{j}={Smin}^{j}+\frac{{I}_{it}^{j}-{min}^{j}\left({I}_{it}\right)}{{max}^{j}\left({I}_{it}\right)-{min}^{j}\left({I}_{it}\right)}({Smax}^{j}-{Smin}^{j})$$

where ${SI}_{it}^{j}$ is the Standardized Indicator *j* for Ecosystem *i* at Year *t,* ${{max}^{j}(I}_{it})$ and ${min}^{j}\left({I}_{it}\right)$ are the maximum and minimum values;${Smax}^{j}$ and ${Smin}^{j}$ are the maximum and minimum standardized values of Indicator *j*, which can be set according to the needs of the specific study^83,84^. They were chosen for clarity in this study so that all *SI*s are on a scale of 1 (*Smin*) to 100 (*Smax*) and thus comparable across indicators.

*I*^*a*^ and *I*^*b*^ are heavily skewed downward, with many small values and few large ones. As a result, arithmetic means are insufficient to describe the “central tendency,” and we instead use the geometric mean. In contrast, because *I*^*c*^ has a more normal distribution, arithmetic means are used. When it comes to calculating global trends, another unique aspect of global research is the enormous variation in the scale of fisheries between countries. The average catch of an EEZ over the study period was used to calculate its weight, ensuring that each country is represented in proportion to the size of its fishery^85^. As a result, in this study, weighted geometric means are used for *I*^*a*^ and *I*^*b*^ and weighted arithmetic means for *I*^*c*^, as shown bellow:

5 $${SI}_{t}^{j}=\prod_{i=1}^{n}{{I}_{it}^{j}{W}_{i}}=\mathrm{exp}\left(\frac{\sum_{i=1}^{n}{W}_{i}ln\left({I}_{it}^{j}\right)}{\sum_{i=1}^{n}{W}_{i}}\right)\mathrm{for }j=a\mathrm{\,and\,}j=b$$

with standard deviation

6 $$\sigma_t^j=\exp{\left(\sqrt{\frac{\sum_{i=1}^{n}{W_i\left(\ln{\left(I_{it}^j\right)}-\bar{\ln{\left(I_t^j\right)}}\right)^2}}{\sum_{i=1}^{n}W_i}}\right)}$$

and

7 $${SI}_{t}^{j}=\frac{\sum_{i=1}^{n}{W}_{i}{I}_{it}^{j}}{\sum_{i=1}^{n}{W}_{i}}\mathrm{for }j=c$$

with standard deviation

8 $${\sigma }_{t}^{j}=\frac{{\sum }_{i=1}^{n}{W}_{i}{({I}_{it}^{j}-{SI}_{t}^{j})}^{2}}{{\sum }_{i=1}^{n}{W}_{i}}$$

where

$${W}_{i}=\frac{{\overline{C} }_{i}}{{\sum_{i}\overline{C} }_{i}}$$

where ${\overline{C} }_{i}$ is the annual average of fish landings over the study period, indicating the size of fisheries in ecosystem *i*.

After normalization, we derived a composite fishing index (*FI*) using their geometric mean

9 $${FI}_{t}={\left(\prod_{j=a}^{c}{SI}_{t}^{j}\right)}^{1/3}$$

and its standard deviation^86^ is

10 $${\sigma }_{t}=\mathrm{exp}\left(\sqrt{{\sum }_{j=a}^{c}{{\sigma }^{2}}_{t}^{j}+\sum_{j}{\sum }_{j\ne k}Cov\left(\ln({I}_{t}^{j}),{ln(I}_{t}^{k})\right)/9}\right)$$

In *FI*, geometric aggregation is used to address concerns about interaction and compensability. It considers the disparities in achievement across multiple indicators^87,88^. Any indicator's poor performance is directly reflected, although not fully compensated for, in the composite index value^88,89^.

To ensure consistency and easy comparison with other global research, we developed these indicators for each of the key 130 EEZ/FAO statistical area ecosystems, then aggregated them by continent and developed or developing countries, following the customary approach of the international community^90^. This classification of countries as developed or developing is based on the Human Development Index^91^. This definition, however, may not be universally accepted.

### Data sources

The following time series data on catch, fishing effort, trophic level, and primary production were compiled:

1. From 1950 to 2017, reported catch statistics were derived from the FAO's global fishery production dataset^92^ and discards and Illegal, Unreported, and Unregulated (IUU) catches from Watson^52^. The FAO catch statistics are generally believed not to include catches that are discarded at sea and taken by IUU fishing. Watson^52^ developed various methods to estimate the IUU catches and discards based on a large number of informal sources such as online, grey country reports, project studies and field surveys and represent the comprehensive data available at the moment. The database^52^ was irregularly updated and publicly accessible at https://doi.org/10.25959/5c522cadbea37).
2. Data on fishing effort (kilowatt-days per year) by country from 1950 to 2019 were supplied by Rousseau et al.^75^. Data on fishing fleets were divided into three categories: unpowered-artisanal, powered-artisanal, and industrial vessels. The fishing effort of unpowered artisanal vessels was evaluated using generic additive models^76,93,94^ based on their catches, daily operation hours, and season length. The effort data are estimates of effective fishing effort standardized for countries, regions, sectors, gear, and years using a set of mathematical models that were first fit to existing data and then extended to countries with missing data or insufficient data to estimate their fishing effort. The technological creep produced by current add-on equipment, electronics, and accessories throughout time was also estimated. The University of Tasmania irregularly published updates to the global effort data^76,93,94^.
3. The Sea Around Us Project^53^ provided data on the mean trophic level (MTL) of the catch from 1950 to 2017. The region-based MTL^81^ measures the mean trophic level of fishery captures from an ecosystem, corrected for potential bias from geographic expansion of fishing, and tracks variations in the mean trophic level of an ensemble of exploited species in response to fishing pressure.
4. The net primary production (PP) by ecosystem was downloaded from Sea Around Us^95^. The PP database are based on a model described by Platt and Sathyendranath^96^. The means are calculated using monthly primary production estimates for the ten-year period 1998–2007 with a spatial resolution of 9 km by the Institute for Environment and Sustainability, EU Joint Research Centre (JRC), Ispra, Italy^95^. The surface area data of each ecosystem was from Ye and Carocci^74^.

It should be mentioned that there is controversy regarding the accuracy of catch counts and estimates^97^. They remain the best data currently available on a worldwide basis^98,99^. The urgent need to build a set of indicators to promote the sustainability of fisheries warrants beginning with the best available data and improving as additional data becomes available.

## Supplementary Information

Supplementary Information.

## Data Availability

All data are available in the main text or the supplementary materials.”
