# Author Correction: A composite fishing index to support the monitoring and sustainable management of world fisheries

**DOI:** 10.1038/s41598-023-38509-8

**Published:** 2023-07-13

**Authors:** Yimin Ye, Jason S. Link

**Affiliations:** 1 Fisheries and Aquaculture Division, Food and Agriculture Organization of the United Nations, Rome, Italy; 2 National Oceanic and Atmospheric Administration, National Marine Fisheries Service, 166 Water Street, Woods Hole, MA 02543 USA

Correction to: *Scientific Reports* https://doi.org/10.1038/s41598-023-37048-6, published online 29 June 2023

The original version of this Article contained an error in the spelling of the author Jason S. Link which was incorrectly given as Jason M. Link.

The original Article and accompanying Supplementary Information file have been corrected.

