# Supplementary material for: A composite fishing index to support the monitoring and sustainable management of world fisheries
Source: Sci Rep. 2023 Jun 29;13:10571. doi: 10.1038/s41598-023-37048-6 (PMC10310702; doi:10.1038/s41598-023-37048-6)
Supplement: Supplementary file 1 — Supplementary Information. [file 41598_2023_37048_MOESM1_ESM.docx]

**Supplementary Materials for**

A composite index of fishing pressure to support the monitoring and sustainable management of world fisheries

Yimin Ye, Jason S. Link

**Statistical information on the validity of the fishing index**

How precisely does the fishing index (*FI*) measure what it intended to assess, namely fishing pressure on the ecosystem? To evaluate its efficacy, we compare the index to the two current indicators: the fish stocks index^1^ (*FSI* ), which measures the proportion of fish stocks within sustainable limits, and the proportion of overfished ecosystems^2^ (*POE*). Despite the fact that these three indices do not measure the exact same metrics of fisheries, *FSI* and *POE* evaluate some components of the fishing index and should therefore have a strong correlation with *FI* assuming *FI* accurately evaluates the performance of fisheries.

*Validation by means of the fish stocks index*

Using the following linear model, the link between the fishing index (*FI*) and the fish stocks index (*FSI*) is described:

$FSI=a+b\times FI$ (1)

where *FSI*≤100 and a and b are coefficients. The outcomes of the regression are summarized in table S1. Both coefficients are extremely significant, and the model explains 89% of the variation in the data (Fig.5). All estimated *FSI* values from the model fall within the 95% confidence interval (Fig.S1).

**Table S1 Compilation of summary statistics for Equation 1**

| Parameter | Estimate | Standard Error | t value | Pr(>\|t\|) |  |
| --- | --- | --- | --- | --- | --- |
| a | 117.589 | 2.963 | 38.331 | 0 | *** |
| b | -0.786 | 0.054 | -12.714 | 0 | *** |
| *Signif. codes: 0 <= '***' < 0.001 < '**' < 0.01 < '*' < 0.05* | | | | | |
| Residual standard error: 2.593 on 19 degrees of freedom | | | | | |
| Multiple R-squared: 0.8948, Adjusted R-squared: 0.8893 | | | | | |
| F-statistic: 161.6 on 19 and 1 DF, p-value: 0.0000 | | | | | |


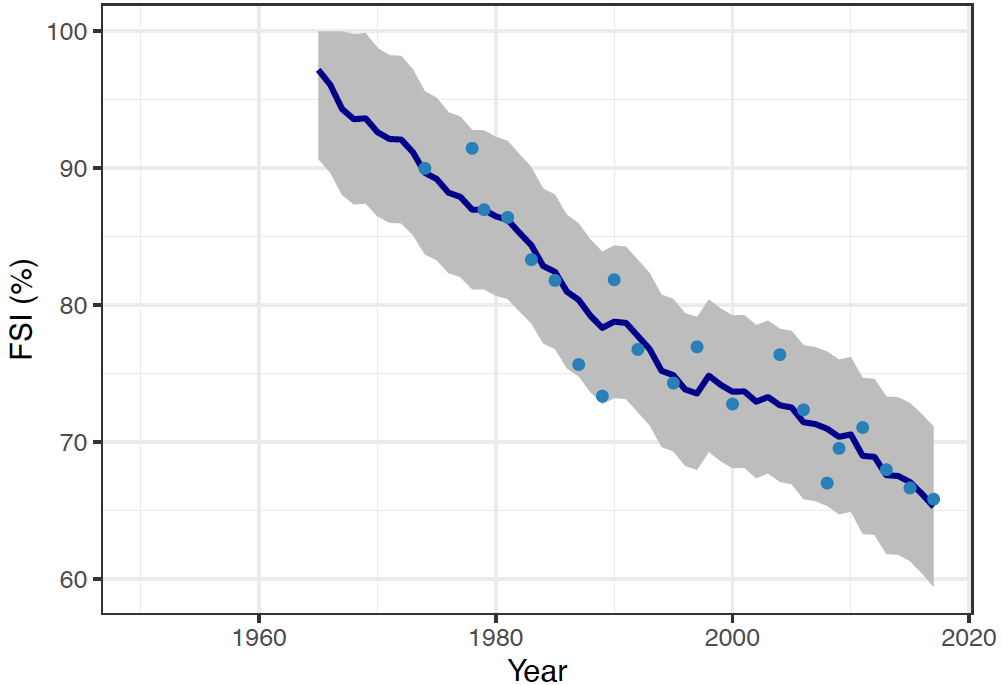


**Fig.S1 Comparison of model predictions to observed data**

*Validation based on the proportion of overfished ecosystems*

The relationship between the fishing index (*FSI*) and the proportion of overfished ecosystems (*POE*) is not linear, and the following nonlinear model is fitted to the data to account for this:

$POE=a(1-e^{b\times FI})$ (2)

where a and b represent coefficients. The statistical overview of the nonlinear fit is shown in table S2. Each coefficient is statistically significant. On this fitted line plot (Fig.6), the regression line closely matches the point's curvature. It appears that there are no systematic departures from the fitted line. The points adequately represent the whole spectrum of predictor values.

However, unlike linear regression, a nonlinear regression equation can take many different forms, and parameter estimations in nonlinear models cannot be interpreted consistently. For nonlinear regressions, *R^2^* and *p* values are invalid. With only one predictor in our nonlinear model, it is prudent to assess the fitted line plot to see the relationship between the predictor and response ^3^. Fig.S2 compares the estimated *FSI* values from the model to the observed values across time. Except for one or two estimates, nearly all estimates fall within the 95% confidence zone.

**Table S2 Compilation of summary statistics for Equation 2**

| Parameter | Estimate | Standard Error | t value | Pr(>\|t\|) |  |
| --- | --- | --- | --- | --- | --- |
| a | 19.938 | 0.494 | 40.35 | 0 | *** |
| b | -0.047 | 0.003 | -15.76 | 0 | *** |
| *Signif. codes: 0 <= '***' < 0.001 < '**' < 0.01 < '*' < 0.05* | | | | | |
| Residual standard error: 1.462 on 66 degrees of freedom | | | | |  |
| Number of iterations to convergence: 13 | | | |  |  |
| Achieved convergence tolerance: 1.32e-06 | | | |  |  |


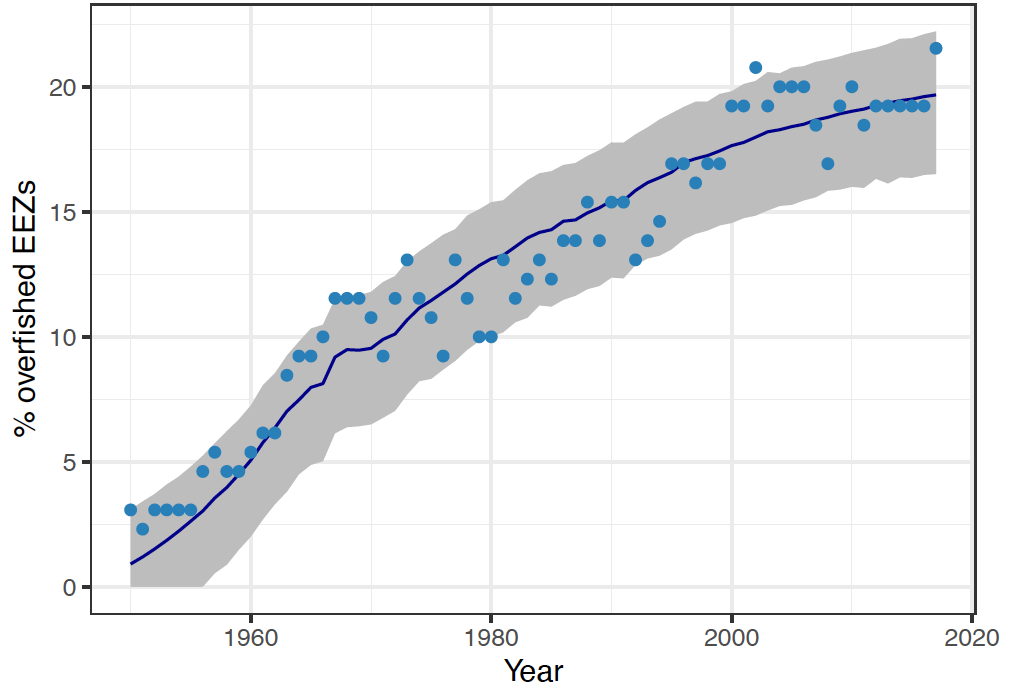


**Fig.S2 Comparison over time between model estimates and observed data**

References

1. FAO. *Fisheries and Aquaculture - Latest publications - Review of the state of world marine fishery resources*. (FAO, Italy, 2011).

2. Link, J. S. & Watson, R. A. Global ecosystem overfishing: Clear delineation within real limits to production. *Sci. Adv.* (2019) doi:10.1126/sciadv.aav0474.

3. Mintab. Data Analysis, Statistical & Process Improvement Tools | Minitab. https://www.minitab.com/en-us/ (2023).
